# Validation and psychometric properties of the Farsi version of the fear of COVID‐19 scale among pregnant women

**DOI:** 10.1002/brb3.2733

**Published:** 2022-08-17

**Authors:** Mojtaba Jafari, Houra Ameri Ahmad, Asra Nassehi, Selman Repisti, Hamid Sharif Nia, Reza Ghanei Gheshlagh

**Affiliations:** ^1^ Student Research Committee, School of Nursing and Midwifery Bam University of Medical Sciences Bam Iran; ^2^ Bahman Hospital Iran University of Medical Sciences Tehran Iran; ^3^ Psychiatric Clinic Clinical Centre of Montenegro Podgorica Montenegro; ^4^ School of Nursing and Midwifery Amol Mazandaran University of Medical Sciences Sari Iran; ^5^ Clinical Care Research Center, Research Institute for Health Development Kurdistan University of Medical Sciences Sanandaj Iran

**Keywords:** fear of COVID‐19, pregnancy, reliability, validity

## Abstract

**Objective:**

The Covid‐19 epidemic, which has become the most challenging issue for health organizations and governments, has led to panic among people, especially pregnant women. The aim of this study was to investigate the psychometric properties of the Persian version of the Fear of COVID‐19 Scale (FCV‐19S) on a sample of Iranian pregnant women.

**Methods:**

This cross‐sectional study was performed on 500 pregnant women referred to gynecology offices in Tehran. Construct validity was performed using exploratory (with maximum likelihood method and Promax rotation) and confirmatory factor analysis. Cronbach's alpha and McDonald omega coefficients were used to examine internal consistency.

**Results:**

The mean age of the women was 28.98 (SD = 5.86) years. In exploratory factor analysis, two factors were extracted—emotional response and physiological response, which together explained 65.21% of the total variance of fear of Covid‐19. In the confirmatory factor analysis, the final model had a good fit: CMIN/d*f* = 1.515, goodness of fit index (GFI) = 0.981, adjusted goodness of fit index (AGFI) = 0.956, normed fit index (NFI) = 0.979, Incremental Fit Index (IFI) = 0.993, comparative fit index (CFI) = 0.993, and root mean square error of approximation (RMSEA) = 0.045 (95% CI: 0.001–0.085). Cronbach alpha and McDonald omega coefficients for the first factor were 0.874 and 0.878, and for the second factor were 0.853 and 0.854, respectively.

**Conclusion:**

The FCV‐19S in pregnant women has a good construct validity and can be used in various studies.

## Graphical Abstract

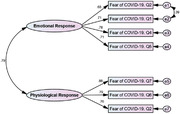

## 1 INTRODUCTION

In December 2019, a viral disease was reported in Wuhan, China, caused by a new, genetically modified virus from the corona virus family called severe acute respiratory syndrome coronavirus 2 (SARS‐CoV‐2), which was named Coronavirus disease 2019 (COVID‐19) (Alipour et al., 2020) and includes fever, cough, and shortness of breath, fatigue, muscle aches, diarrhea, sore throat, loss of sense of smell, abdominal pain, and respiratory failure (Aliakbari Dehkordi et al., 2020). The virus is transmitted from human to human and animal, and has an average incubation period of 5 days (Sadeqi et al., 2020). COVID‐19 disease in Iran was first reported in Qom on February 20, 2020, and so far, the Iranian people have experienced different waves of this epidemic, which is associated with high mortality (Peykari et al., 2020).

Due to its high transmissibility, it spread to many countries in a short time (Shahyad & Mohammadi, 2020). Following the outbreak, as there was still no vaccine or treatment for COVID‐19, governments focused on public health measures such as social distancing and the use of masks (Zhu et al., 2020). These actions increased fear and stress, so that in some countries people bought and stored food, face masks, and even weapons (Abuhammad et al., 2020; Bakioğlu et al., 2020; Skoda et al., 2020). However, despite vaccination, the fear of COVID‐19 still persists. Unprecedented measures such as border control and quarantine were also implemented (Ahorsu et al., 2020; Alyami et al., 2020).

Fear is an unpleasant emotional state that is triggered in response to threatening stimuli. Fear and anxiety about possible infection can be destructive and lead to mental disorders and stress (Rahmanian et al., 2020). The experience of SARS and Ebola pandemic has also shown that fear of a pandemic exacerbates the damage caused by the disease; so, controlling the social response is one of the essential steps in dealing with pandemics (García‐Reyna et al., 2020).

Pregnant women are one of the groups at a higher risk for COVID‐19 disease (Riahy, 2020). Increased levels of anxiety and stress during pregnancy increase the risk of eclampsia, gestational depression, nausea and vomiting, preterm delivery, low birth weight, and even low Apgar scores (5). High levels of fear may prevent a person from making rational decisions in response to illness. For example, some pregnant women did not go to health centers for routine screening during pregnancy for fear of developing COVID‐19. Some pregnant women have even requested termination of pregnancy and cesarean section due to high fear and anxiety (Rashidi Fakari & Simbar, 2020). Also, despite the risks of self‐medication during pregnancy, some pregnant women have become obsessed with using the drugs listed in the COVID‐19 guidelines, such as hydroxychloroquine (Banerjee, 2020). Therefore, to reduce potential physical and mental problems in pregnant women, it is necessary to assess the level of fear associated with COVID‐19.

A valid, reliable, and concise instrument is needed to measure the fear of COVID‐19 in pregnant women. Fear of COVID‐19 Scale (FCV‐19S) is one of the tools used to measure the fear of COVID‐19 (Ahorsu et al., 2020), which has been translated into many languages (in more than 30 countries), and its psychometric properties have been reported in different populations (Alyami et al., 2020; Bitan et al., 2020; Caycho‐Rodriguez et al., 2020; Elemo et al., 2020; Huarcaya‐Victoria et al., 2020; Masuyama et al., 2020; Pang et al., 2020; Perz et al., 2020; Sakib et al., 2020; Soraci et al., 2020). Given the consequences of the irrational fear of COVID‐19 among pregnant women, it seems necessary to measure this concept with a valid and reliable tool whose psychometric properties have been confirmed. Accordingly, this study was performed with the aim of determining validity and internal consistency of the Persian version of the FCV‐19S in Iranian pregnant women.

## 2 MATERIALS AND METHODS

### 2.1 Participants and setting

This cross‐sectional study was performed in December 2021 on 500 pregnant women referring to gynecological offices in the south of Tehran (capital of Iran). In this area, four offices of gynecologists were purposefully selected because they had the most clients. Women referring to these offices were selected by convenience sampling. Four trained nursing students were used to collect data. To perform exploratory factor analysis (EFA), 5–10 samples are required per item. Some researchers also consider the sample size of 200–300 people to be appropriate and 300–500 people to be excellent. It is also recommended that the sample size should not be less than 200 to perform the confirmatory factor analysis (CFA) (Plichta et al., 2013). The participants were randomly divided into two halves. The EFA was performed on 250 people and the confirmatory factor analysis was performed on the rest of the samples. The inclusion criteria were the following: (a) age ≥18 years, (b) lack of any high‐risk pregnancy complication, (c) voluntary participation, and (d) ability to read and write. Exclusion criteria were incomplete completion of questionnaires.

After coordination with gynecologists, explaining the objectives of the study to pregnant women, and obtaining informed written consent, the questionnaires were distributed in person. Anonymity was guaranteed to participants as well as confidentiality of the collected data. In addition, the participants were told that answering the questionnaires is optional and has no effect on their treatment process. Participants were selected by multi‐stage cluster sampling. At first, each area of Tehran was considered as a cluster, and then four clusters were randomly selected from the clusters and pregnant women referring to the offices of each cluster were selected by convenience sampling.

### 2.2 Translation

After obtaining permission from the original designer, the English version of the fear of COVID‐19 was translated to Persian through backward and forward translations by two independent bilingual translators. Any differences between the two versions were discussed and resolved by the research team. In the next step, the final Persian version was translated into English by two other translators independently (Beaton et al., 2000).

### 2.3 Measure

Demographic information form and Fear of COVID‐19 Scale (FCV‐19S) were used to collect data.

#### 2.3.1 Demographic information form

The demographic form included information such as participants’ age, gestational age, number of children, education, occupation, family history of COVID‐19, number of previous pregnancies, comorbidity, and general health status. Comorbidity meant any illness for which a person was taking medication. General health was evaluated with only one question that had three answers (good, moderate, poor): In general, how is your general health?

#### 2.3.2 Fear of COVID‐19 scale (FCV‐19S)

The FCV‐19S is a unidimensional scale that measures people's fear of the COVID‐19 and was designed by Ahorsu et al. (2020). The FCV‐19S has 7 items that are scored based on a 5‐point Likert scale from 1 (strongly disagree) to 5 (strongly agree). The minimum score possible for each question is 1, and the maximum is 5. The total score is calculated by adding the scores of each item; thus, the total scores can vary between 7 and 35. Higher scores indicate greater fear of the COVID‐19. The preparation of the original version was done in several steps. First, by extensive literature review, all general scales of fear were collected and based on them, a pool item (including 28 items) was formed, which was evaluated in terms of content by experts. Eleven items were removed and the remaining 17 were sent to a different panel for review. Seven items were also removed based on the opinions of the second panel of experts. Finally, the remaining 10 items were tested on 46 subjects for initial scale evaluation. A four‐point Likert scale was used to test whether individuals understood item descriptions. In factor analysis, 3 items were deleted and the final 7 items remained.

### 2.4 Face and content validity

In scale design, after preparing the item pool, face and content validity are assessed quantitatively. In face validity, impact score is evaluated, and in content validity, CVR (content validity ratio) and CVI (content validity index) are evaluated, and based on the score of the items, the researcher decides to remove or keep them. In psychometric evaluation, the researcher is not permitted to delete or add any item to the original version of the questionnaire, and rewrites the items for better understanding only on the advice of the subjects (qualitative face validity) and experts (qualitative content validity). Therefore, the researcher is allowed to rewrite the items for better understanding based on the recommendations of the subjects (qualitative face validity) and experts (qualitative content validity) (Polit, 2015). In order to check the face validity, the Persian version of this tool was given to 5 pregnant women to review it in terms of writing, meaning, and ambiguity and give us feedback. After reviewing the suggestions and making the recommended changes, the final version was given to 5 experts (gynecologist, psychiatrist, psychologist, nurse, and methodologist) to evaluate it in terms of content.

### 2.5 Statistical analysis

IBM SPSS ver. 25 and IBM SPSS Amos programs were used for data analysis. Univariate and multivariate data distributions were evaluated to investigate the normality and outliers. Multivariate normality was assessed using the Mardia's coefficient of multivariate kurtosis. A Mardia's coefficient greater than 8 was an indication of deviation in kurtosis (Esposito et al., 2020). Exploratory factor analysis (EFA) with maximum likelihood method and Promax rotation was used to explore the factor structure of FCV‐19S. There is no general rule about sample size for EFA; however, a sample size of between 200 and 300 is considered appropriate (Arrindell & Van der Ende, 1985; Comrey, 1988). Also, according to the rule of thumb, 10 subjects per item are considered suitable for EFA (Cabrera‐Nguyen, 2010). For CFA, the sample size should not be less than 200 people (Ho, 2013).

**FIGURE 1:**
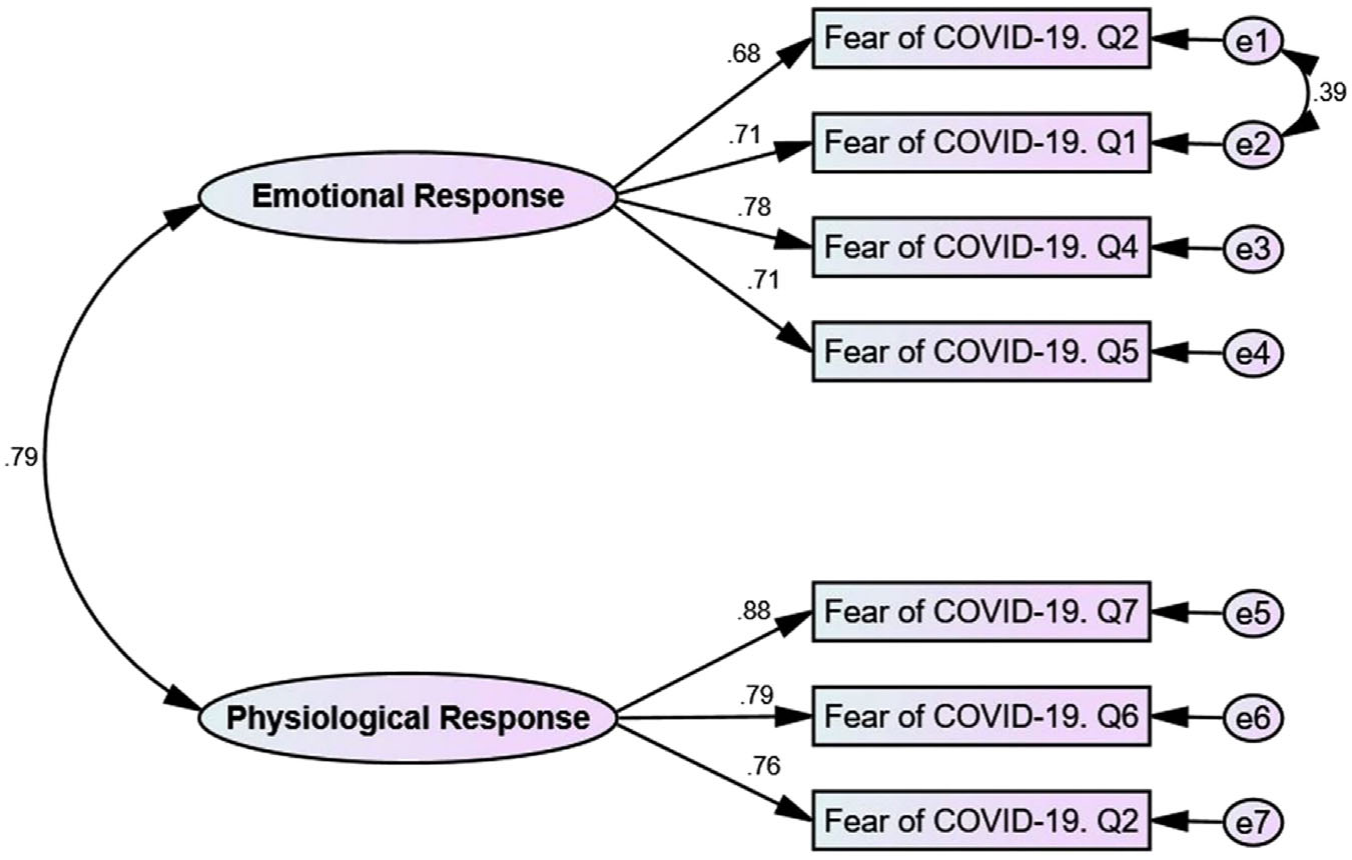
Confirmatory factor analysis of FCV‐19S in Iranian pregnant women

Data are suitable for factor analysis if the Kaiser‐Meyer‐Olkin (KMO) index is above 0.6, the Bartlett test of sphericity is significant, in the correlation matrix the coefficients are 0.3 and higher, and the communalities are above 0.3 (Burton et al., 2019; Field, 2013). The number of factors was determined based on parallel analysis. Only items with a factor loading of 0.30 or higher were retained. We used CFA to confirm the latent structure of the instrument. The model fit was examined using fit indices, such as chi‐squared test (*χ^2^*), chi‐square ratio to degree of freedom (CMIN/df), goodness of fit index (GFI), adjusted goodness of fit index (AGFI), normed fit index (NFI), incremental fit index (IFI), confirmatory fit index (CFI), and root mean square error of approximation (RMSEA). GFI, AGFI, NFI, CFI and IFI indices should be above 0.9 whereas RMSEA has to be less than 0.05 (Bentler, 1990). Cronbach's alpha and McDonald omega coefficients were used to evaluate the internal consistency, wherein values above 0.7 are acceptable (Ebadi et al., 2019; Sharif Nia et al., 2019). Due to the lack of re‐access to these samples, the stability of the questionnaire was not evaluated.

### 2.6 Ethical consideration

All procedures performed in studies involving human participants were in accordance with the ethical standards of the institutional and/or national research committee and with the 1964 Helsinki declaration and its later amendments or comparable ethical standards. This study was approved by the ethics committee of Bam University of Medical Sciences (IR.MUBAM.REC.1400.057) and informed consent was obtained from all individual participants included in the study. Women were informed about the goals and methods of the study. Questionnaires were also distributed anonymously among the participants.

## 3 RESULTS

### 3.1 Findings description

The participants’ mean age was 28.98 years (SD = 5.86) and the mean gestational age was 25.52 (SD = 9.34) weeks. The age range of participants was 16–45 years and their gestational age range was 4–41 weeks. The majority of women were housewives (74.6%) and had university education (46%). About 40.4% of pregnant women reported that at least one member of their family has ever had COVID‐19. Also, 77.6% of women did not have comorbidities. In terms of general health, 15% of the samples reported excellent health, 52.8% good, 31.4% moderate, and 0.8% bad (Table 1). For the total sample, the mean value of the FCV‐19S was 22.21 (SD = 5.66), with a range of 7–35.

**TABLE 1:** Participant demographics (N = 500)

Demographics		*n*	(%)
literacy	Primary school	63	12.6
	Secondary & high school	207	41.4
	University	230	46
Job	Employment	127	25.4
	Unemployment	373	74.6
General Health	Excellent	75	15
	Good	264	52.8
	Moderate	157	31.4
	Poor	4	0.8
Family history of COVID‐19	Yes	202	40.4
	No	298	59.6
Number of previous pregnancies	0	141	28.2
	1 or more	359	71.8
Gestational age	First trimester	51	10.2
	Second trimester	210	40.2
	Third trimester	239	40.6
Comorbidity	Yes	112	22.4
	No	388	77.6

### 3.2 Face and content validity results

In the face validity, item #6 (I cannot sleep because I'm worrying about getting coronavirus‐19) was rewritten and modified as follows: “Worrying about illness has made it impossible for me to sleep as before.” No changes were recommended in the content validity due to the short and simple items.

### 3.3 Construct validity results

The KMO index was 0.785 and Bartlett test of sphericity indicated that the data were appropriate for the factor analysis (*χ^2^* = 1815.425, p = 0.001). In the EFA, two factors were extracted that together explained 65.21% of the total variance of fear of COVID‐19. The first factor had four items (1, 2, 4, and 5) that explained 36.94% of the total variance and focused on emotional responses. The second factor had three items (3, 6, and 7) that explained 28.27% of the total variance and referred to physiological responses (Table 2).

**TABLE 2:** Exploratory factor analysis for the Persian version of fear of COVID‐19 scale (FCV‐19S)

Factors	Items	Factor loading	Communality (h^2^)	Eigenvalue	% of variance	Internal consistency
Emotional responses	Item 1	0.911	0.791	2.586	36.94	α = 0.874, Ω = 0.878
	Item 2	0.884	0.756			
	Item 4	0.747	0.616			
	Item 5	0.646	0.482			
Physiological responses	Item 3	0.871	0.782	1.979	28.27	α = 0.853Ω = 0.854
	Item 6	0.805	0.626			
	Item 7	0.757	0.590			

The results of the CFA showed that the two‐factor structure of the FCV‐19S fitted well with the data (CMIN/df = 1.515, Goodness of Fit Index (GFI) = 0.981, Adjusted Goodness of Fit Index (AGFI) = 0.956, Normed Fit Index (NFI) = 0.979, Incremental Fit Index (IFI) = 0.993, Comparative Fit Index (CFI) = 0.993, and Root Mean Square Error of Approximation (RMSEA) = 0.045 (95% CI: 0.001‐0.085) (Figure 1).

### 3.4 Reliability

The results of internal consistency showed that Cronbach's alpha and McDonald omega for the two extracted factors were above 0.8, which is acceptable.

## 4 DISCUSSION

The results of this study, which aimed to investigate the psychometric properties of the Persian version of the FCV‐19S on pregnant women, showed that this scale has very good internal consistency reliability, which is consistent with the findings of previous studies in other countries (Alyami et al., 2020; Bitan et al., 2020; Caycho‐Rodriguez et al., 2020; Elemo et al., 2020; Huarcaya‐Victoria et al., 2020; Masuyama et al., 2020; Pang et al., 2020; Perz et al., 2020; Sakib et al., 2020; Soraci et al., 2020). Additionally, these results demonstrated that this scale has a two‐factor structure. The results of studies conducted to evaluate the psychometric properties of Japanese and Russian versions of the FCV‐19S showed that this scale has a single‐factor structure (Masuyama et al., 2020; Reznik et al., 2020). In the current study, the extracted factors explained 65.21% of the total variance of this concept, which is consistent with the results of studies conducted in the United States, Turkey, and Brazil (Cavalheiro & Sticca, 2020; Özdeni & Aktura, 2020; Perz et al., 2020). In the study by Martínez‐Lorca et al. (2020), the Spanish version of the FCV‐19S had a single‐factor structure that explained less than 50% of the total variance .

The results of CFA showed that the two‐factor model has the best fit. While in the Bangladeshi (Sakib et al., 2020), Italian (Soraci et al., 2020), Turkish (Satici et al., 2020), and Arabic (Alyami et al., 2020) versions, the one‐factor model had a good fit. The reason for this finding can be attributed to the differences in the cultural characteristics and context of the studied communities as well as their perception of fear of COVID‐19. On the other hand, these studies have all been performed on the general population, while the present study was conducted exclusively on pregnant women. All studies in this field have shown that this scale has at least satisfactory psychometric properties. Because COVID‐19 is a global epidemic (that is, a pandemic), it seems that people of all ages feel this threat and fear almost identically, so they might answer questions similarly (Sakib et al., 2020).

The results of various studies showed that the factor loading of items #3, #6, and #7 that focus on physiological responses is lower than of other items (Alyami et al., 2020; Masuyama et al., 2020; Reznik et al., 2020). Given that these items describe symptoms such as hand sweating, sleep disturbance, and palpitations due to COVID‐19, it can be concluded that somatic symptoms caused by fear are less experienced by participants (Masuyama et al., 2020); instead, the factor loading of the emotional response factors was higher than the physiological response. Extreme fear of the COVID‐19 epidemic had led to many psychological problems such as anxiety, anger, post‐traumatic stress and confusion, and even suicide (Goyal et al., 2020; Haktanir et al., 2020; Luo et al., 2021; Mamun & Griffiths, 2020a). In Bangladesh, for example, a person committed suicide just by thinking that his fever and cold symptoms were related to the COVID‐19, while an autopsy showed that he was healthy (Mamun & Griffiths, 2020b). The highest mean score obtained in this study was related to item #3 (3.38), while in the systematic review and meta‐analysis of Luo et al. (2021), Item #1 (3.32) and #3 (1.78) had the highest and lowest scores, respectively. Also, the mean score of fear of COVID‐19 in this study was 22.21, which was higher than the global average (18.57). The reason for this finding can be attributed to the pregnant woman's concerns about the health of the baby, which can intensify their fears in this epidemic. The results showed that the reliability of the FCV‐19S in pregnant women has an acceptable level, which is also consistent with the results of the original study (Ahorsu et al., 2020).

Given that different groups of people perceive the fear of COVID‐19 in different ways, it is necessary to examine the psychometric properties of this scale. One of the strengths of this study was the comprehensive and accurate evaluation of some main psychometric properties of this instrument in pregnant women because they may perceive the fear of COVID 19 differently from others due to their special characteristics. One of the main limitations of this study was that the stability (i.e., test–retest reliability) of this tool was not investigated. Due to the occurrence of various waves of COVID 19 epidemic in Iran and frequent closures, it was not possible to access the same sample again. However, this limitation could be regarded as a recommendation for future research in this field. The next limitation was that the participating pregnant women were available.

Given the concerns that pregnant women have about their own health and that of their child, as well as the consequences of extreme fear, it is recommended that in pandemic waves, the fear status of pregnant women attending offices be assessed based on this scale. Based on the results, give them the necessary training.

### 4.1 Implications

It seems that extreme fear can lead to aggravation of physical symptoms and even the occurrence of inappropriate behaviors. Therefore, measuring the fear of COVID‐19 in pregnant women is essential. The evolving nature of the COVID‐19 and the public health measures proposed to reduce the spread of the disease, such as quarantine, isolation, and physical distancing, inadvertently led to increased fear among the population (Wastnedge et al., 2021). Infectious diseases can also cause severe fear in pregnant women, as complications and mortality often increase in pregnant women. Recent outbreaks of infectious diseases such as Zika virus and Acute Respiratory Syndrome (SARS) have led to severe birth defects and poor birth outcomes (e.g., preterm labor), respectively (Marrs et al., 2016; Wong et al., 2004). Despite the unintended consequences of increased fear, fear is an adaptive feeling that protects the individual from potential threats and increases the willingness to engage in health‐promoting activities that help reduce the prevalence of the disease (e.g., regular hand washing, conscious physical distance) (Corbett et al., 2020; Pakpour & Griffiths, 2020).

## 5 CONCLUSION

The present study showed that the Persian version of FCV‐19S in pregnant women has at least satisfactory psychometric properties and can be used in various studies.

## CONFLICT OF INTEREST

The authors declare no competing interests.

## AUTHOR CONTRIBUTIONS

Houra Ameri Ahmad & Mojtaba Jafari: data collection and manuscript preparation; Reza Ghanei Gheshlagh & Asra Nassehi: manuscript preparation and study conceptualization; Mojtaba Jafari & Reza Ghanei Gheshlagh: study design; Selman Repisti: final revision and grammar editing; Hamid Sharif Nia: statistical analysis.

### PEER REVIEW

The peer review history for this article is available at: https://publons.com/publon/10.1002/brb3.2733.

## Data Availability

The datasets used and/or analyzed during the current study are available from the corresponding author on reasonable request.
